# E-Learning per Webinar in der Orthopädie und Unfallchirurgie: Intensiviertes Angebot und gesteigerte Nachfrage in der COVID-19-Pandemie

**DOI:** 10.1007/s00113-022-01245-1

**Published:** 2022-10-14

**Authors:** Felix Erne, David A. Back, Tobias Gehlen, Heiko Baumgartner, Alexander Zimmermann, AG Digitalisierung der DGOU, Ricarda J. Seemann

**Affiliations:** 1 Siegfried Weller Institut für unfallmedizinische Forschung Tübingen, Klinik für Unfall- und Wiederherstellungschirurgie, Berufsgenossenschaftliche Unfallklinik Tübingen, Eberhard Karls Universität Tübingen, Schnarrenbergstraße 95, 72076 Tübingen, Deutschland; 2 Klinik für Unfallchirurgie und Orthopädie, Bundeswehrkrankenhaus Berlin, Berlin, Deutschland; 3 Centrum für Muskuloskeletale Chirurgie, Charité – Universitätsmedizin Berlin, Berlin, Deutschland; 4 Geschäftsstelle Deutsche Gesellschaft für Orthopädie und Unfallchirurgie (DGOU), Berlin, Deutschland

**Keywords:** Webinar, Fortbildung, Digitalisierung, Online, COVID-19-Pandemie, Webinar, Education, Digitalization, Online, COVID-19 pandemic

## Abstract

**Hintergrund:**

Im Verlauf der COVID-19-Pandemie haben offizielle Schutzmaßnahmen traditionelle Präsenzfortbildungen zum Erliegen gebracht. Für das Fach Orthopädie und Unfallchirurgie (O&U) gibt es bisher bezüglich der Angebots- und Nachfragesituation von E‑Learning per Webinar im Kontext der COVID-19-Pandemie keine belastbaren Zahlen.

**Fragestellung:**

Das Ziel der vorliegenden Arbeit ist die quantitative Beschreibung von Angebot und Nachfrage deutschsprachiger Online-Fortbildungen mit Webinar-Charakter aus der Orthopädie und Unfallchirurgie (O&U) im zeitlichen Zusammenhang mit der COVID-19-Pandemie.

**Material und Methoden:**

Über gängige Suchmaschinen wurden deutschsprachige, nichtkommerzielle, editierte und wissenschaftlich fundierte Fortbildungen in Form von Webinaren im Bereich O&U von Anbietern mit Sitz in Deutschland identifiziert und interviewt.

**Ergebnisse:**

Alle 4 eingeschlossenen Anbieter (AO Online Campus, BVOU Study Club, OU TO GO, WebDGU) nahmen an den strukturierten Interviews teil und stimmten einer Offenlegung der Teilnehmerzahlen zu. Das Angebot von Webinaren stieg im zeitlichen Zusammenhang mit der COVID-19-Pandemie an. Ebenfalls erhöhten sich bei allen 4 Anbietern die Teilnehmerzahlen.

**Diskussion:**

Während OU TO GO und der BVOU Study Club bereits vor der Pandemie auf E‑Learning-Formate spezialisiert waren, wurde das Kursangebot der AO durch den neu etablierten AO online Campus erweitert und WebDGU ganz neu konzipiert. Eine Limitation der Studie ist die exklusive Ausrichtung auf Webinare und nichtkommerzielle Anbieter. Die Ergebnisse lassen auf positive Entwicklungen in O&U im Bereich E‑Learning hoffen.

**Graphic abstract:**

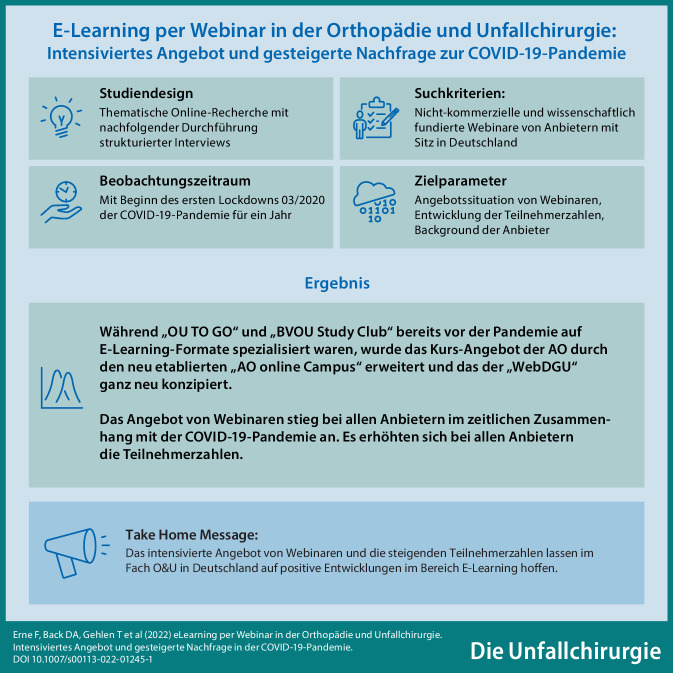

## Abstract (en)

**Background:**

Contact teaching was prohibited by nationwide lockdowns during the COVID-19 pandemic. The scientific literature contains no concrete figures concerning e‑learning via webinars in the subject of orthopedics and trauma surgery in the context of the COVID-19 pandemic.

**Objective:**

This research study was established to collect facts and figures about webinars as a representative part of e‑learning in the subject of orthopedics and trauma surgery in the context of the COVID-19 pandemic.

**Material and methods:**

German-speaking non-commercial and edited webinars, produced by suppliers from Germany were identified using common search engines. Structured interviews with questionnaires about provider, format, and number of participants in the webinars over time, were offered to the operating companies.

**Results:**

The study included four suppliers of webinars (AO Online Campus, BVOU Study Club, OU TO GO and WebDGU). There was approval of all operating companies to participate in the interviews and to disclose facts and figures about their webinars. All suppliers showed an increased supply and demand for webinars during the COVID-19 pandemic.

**Discussion:**

OU TO GO and BVOU Study Club already offered webinars prior to the COVID-19 pandemic. AO online Campus was designed in addition to the existing portfolio. The supply of WebDGU was newly established. Limitations of this study are the exclusion of commercial suppliers, non-German operating companies and all other types of e‑learning except webinars. The increased supply and demand for webinars during the COVID-19 pandemic gives hope for further improvement of e‑learning in the subject of orthopedics and trauma surgery.

## Einleitung

Im Verlauf der COVID-19-Pandemie haben offizielle Kontaktbeschränkungen die Arbeitswelt grundlegend verändert und mit ihr die Fort- und Weiterbildungen in Präsenz zum Erliegen gebracht [1]. Als Reaktion wurden etablierte Lernformate digitalisiert, Kursmodelle zeitlich und inhaltlich angepasst, aber auch neuartige Konzepte entwickelt [2–4]. Bisher galt der Fortschritt der Digitalisierung in der Gesundheitsbranche im internationalen Vergleich eher als behäbig [5]. Durch die COVID-19-Pandemie wird ein positiver, beschleunigender und nachhaltiger Einfluss auf die Digitalisierung erwartet [6–8]. Der Begriff E‑Learning ist nicht einheitlich definiert und wird für viele Lernformate verwendet. Hierzu zählen u. a.:

- Web-based Trainings: Lerninhalte werden in der Regel in Folienform aufgearbeitet und ggf. durch ein Quiz abgefragt. Das Format ist v. a. für innerbetriebliche Schulungen beliebt.
- Videokurse: Das Wissen wird in Form von vorgefertigten Videos angeboten und meist durch ein Quiz abgefragt. Das Format ist bei kommerziellen Schulungen beliebt.
- Webinare: Der Vortrag eines Referenten wird live angeboten; das Format ist in der Regel interaktiv gestaltet. Das Format ist für wissenschaftliche Vorträge und Fortbildungen beliebt.
- Virtual Classrooms: Die Interaktion einer kleinen Anzahl von Teilnehmern unter Anleitung eines Dozenten dominiert diese Unterrichtsform. Die gemeinsame Diskussion steht hier im Vordergrund. Das Format ist für kreative Gruppenarbeiten beliebt.

Darüber hinaus gibt es Vermittlungsplattformen, welche Bildungsinhalte in freier oder fachlich editierter Form anbieten. Dazu gehören Wiki- oder Videoplattformen, Social-Media-Kanäle und mobile Apps. Die Angebote von Virtual Reality, Augmented Reality oder sog. Serious Games spielen bislang eine eher untergeordnete Rolle [5]. Die Finanzierung kann über Spenden, ehrenamtliches Engagement, Fach- und Interessenverbände, Pay-per-Use- und Abonnementmodelle, Provisionen oder Werbung realisiert werden. Für das Fach Orthopädie und Unfallchirurgie (O&U) gibt es bezüglich des Angebots und der Nachfrage von E‑Learning im Kontext der COVID-19-Pandemie bisher keine belastbaren Zahlen. Eine wissenschaftliche Begleitung dieser Entwicklung ist jedoch wünschenswert und erforderlich, um für zukünftige Herausforderungen gewappnet zu sein. Ziele der vorliegenden Arbeit sind die Dokumentation der aktuellen Entwicklung für O&U in Deutschland anhand der dargebotenen Webinare zu Beginn der Pandemie sowie eine Analyse der Entwicklung von Angebot und Nachfrage anhand der Teilnehmerzahlen.

## Material und Methoden

Um in den zahlreichen und uneinheitlichen Formen des E‑Learning eine standardisierte Vergleichbarkeit gewährleisten zu können, wurde das „Webinar“ als repräsentatives Merkmal für die abzubildende Entwicklung definiert. Das Webinar ist ein weit verbreitetes und anerkanntes Lernformat. Der organisatorische und inhaltliche Aufbau entspricht weitgehend dem einer akademischen Lehrveranstaltung, einer wissenschaftlichen Präsentation oder einer klinischen Fortbildung. Diese traditionellen Lernformate sind aus dem medizinischen Arbeitsumfeld kaum wegzudenken und erfreuen sich einer großen Beliebtheit. Das Webinar erzeugt als digitales Lernformat wenig Berührungsangst und eine hohe Akzeptanz. Durch die sprunghaft ansteigende Verfügbarkeit von Konferenz-Software ist die technische Umsetzung sehr einfach. Des Weiteren bietet sich der Vorteil einer klaren messbaren zeitlichen und inhaltlichen Begrenzung. Aus diesem Grunde wurde aus dem vielfältigen und sich rasch verändernden Angebot von digitalen Lerninhalten im Rahmen dieser Arbeit das Webinar als Untersuchungsgegenstand ausgewählt.

Der zu untersuchenden Zeitraum wurde auf März 2020 bis März 2021 festgelegt. Der Hintergrund ist, dass die ersten Erkrankungsfälle von COVID-19 in Deutschland im Januar 2020 auftraten. Ab dem 22.03.2020 trat auch der erste sog. Lockdown in Kraft. In den folgenden Monaten wurden weitere Coronaverordnungen verabschiedet, welche die persönlichen Kontakte beschränkten. Am 04.03.2021 wurde der Stufenplan für die Coronalockerungen vorgelegt und schrittweise umsetzt. Somit ist davon auszugehen, dass der zu untersuchende Zeitraum die sich früh auf dem Markt befindlichen Anbieter abbildet.

Um relevante Anbieter auflisten zu können, wurde über die gängigen Suchmaschinen Google, Startpage, Bing, Yahoo, DuckDuckGo und Fireball eine Recherche zu Angeboten für E‑Learning im Fachgebiet O&U durchgeführt. Als Suchbegriffe wurden „Orthopädie“ und/oder „Unfallchirurgie“ und „eLearning“ und/oder „Webinar“ und/oder „Onlinefortbildung“ festgelegt.

Als standardisierte Einschlusskriterien wurde folgendes idealisiertes Anforderungsprofil für die Zwecke einer fachspezifischen Fortbildung der O&U in Webinar-Form auf die gefunden Anbieter angewendet:

- Lehrangebot online verfügbar,
- Lehrangebot als regelmäßiges wiederkehrendes Webinar verfügbar,
- Lehrangebot in deutscher Sprache verfügbar,
- Sitz der Geschäftsstelle laut Impressum in Deutschland,
- Geschäftsmodell nichtkommerziell ausgerichtet,
- Lehrinhalte exklusiv aus dem Fachgebiet O&U,
- Lehrinhalte haben innovativen Charakter und relevanten klinischem Praxisbezug,
- Qualitätssicherung durch editierte Inhalte unter Einhaltung wissenschaftlicher Standards.

Explizit ausgeschlossen wurden:

- Anbieter von Zertifizierungsmaßnahmen zur Positionierung am Gesundheitsmarkt,
- Lehrangebot für Studierende,
- Ausrichtung auf CME-Zertifizierung,
- Vermittlungsplattform für fremde Lehrangebote, kein eigenständiger Anbieter.

Es wurden potenzielle Messparameter zusammengetragen und bezüglich ihrer Verfügbarkeit und Aussagekraft bewertet. Klassische Kennzahlen von E‑Commerce-Plattformen können nicht auf Webinare angewendet werden. Die Offenlegung klassischer betriebswirtschaftlicher Kennzahlen durch die Anbieter wird nicht erwartet. Die Abfrage von Angebot und Nachfrage kann gut gemessen werden. Die Auswertung von Evaluationen ist geeignet, um das Feedback der Teilnehmer bewerten zu können.

Es wurde ein standardisierter Fragebogen entworfen, um die detektierten Betreiber der Plattformen kontaktieren und befragen zu können. Die Befragung wurde als strukturiertes Interview mit 3 Fragenkomplexen konzipiert:

- Informationen zum Anbieter:
  - Unternehmensstruktur,
  - Gewinnbestreben,
  - Finanzierung,
  - Spezialisierung auf Zielgruppen.
- Informationen zum Format:
  - Abfrage der angebotenen Formate,
  - Umfang der interaktiven Inhalte,
  - Ablauf des Anmeldungsprozesses,
  - Notwendigkeit einer Registrierung,
  - entstehende Kosten,
  - Notwendigkeit einer Mitgliedschaft,
  - Möglichkeit der Erlangung von CME-Punkten,
  - Vorhandensein eines Archivs mit flexiblem Abruf der Inhalte.
- Informationen zu Kennzahlen:
  - Abfrage der Termine der Webinare,
  - Abfrage der jeweiligen Teilnehmerzahlen der Webinare.

Die Auswertung der strukturierten Interviews erfolgte deskriptiv. Die Speicherung, Auswertung und Visualisierung der gesammelten Daten erfolgte mittels Excel (Fa. Microsoft Inc., Redmond, WA, USA).

## Ergebnisse

Gemäß den oben genannten Kriterien wurden in alphabetischer Reihenfolge folgende Anbieter identifiziert:

- AO Trauma Deutschland online Campus (AO online Campus), https://aotrauma.meet-online.net/, von AO Trauma Deutschland (Berlin),
- BVOU Study Club, https://www.bvoustudyclub.net/, von Berufsverband für Orthopädie und Unfallchirurgie e. V. (Berlin),
- Orthopädie und Unfallchirurgie TO GO (OU TO GO), https://ou.medizintogo.de/, von MEDIZIN TO GO gemeinnützige GmbH (Bochum/Berlin),
- Web-Seminar-Reihe der DGU (WebDGU), https://www.bikmed.de/, von bikmed – Bildungsinstitut für Kompetenz in der Medizin GmbH der Akademie der Unfallchirurgie GmbH (München), das Portal wurde mit dem Kursportal der AUC verschmolzen, https://fortbildung.auc-online.de/.

**Figure 1:**
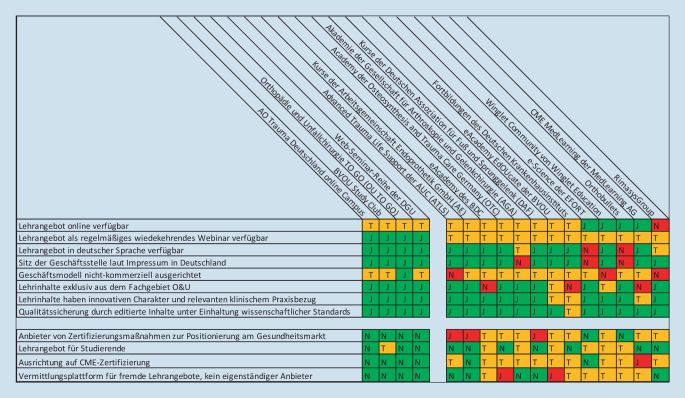

Alle Betreiber erklärten sich bereit, im Rahmen des strukturierten Interviews Informationen über ihr Angebot zur Verfügung zu stellen, sowie detaillierte Daten zu den Teilnehmerzahlen ihrer Webinare anzugeben. Die Offenlegung betriebswirtschaftlicher Kennzahlen wurde nicht gewünscht. Etwaige Evaluationen wurden ausdrücklich nur für die interne Qualitätssicherung und Bewertung von Referenten durchgeführt. Die Ergebnisse der Befragung sind in Abb. 1 dargestellt.

### AO online Campus

Der AO online Campus wurde im Frühjahr 2020 ins Leben gerufen, da die geplanten Präsenzfortbildungsveranstaltungen der AO Trauma pandemiebedingt als nichtrealisierbar eingeschätzt wurden. Die AO-Trauma-Basisseminare 1–3 wurden als Hybridveranstaltung neu konzipiert und der Seminarteil für die registrierten Teilnehmer als abendliche 60- bis 90 minütige Live-Webinare im mehrtägigen Abstand abgehalten. Ab Herbst 2020 kamen die AO-Trauma-Spezialkurse zusätzlich als Webinare ins Angebot. Es werden Live-Inhalte vermittelt und vertiefend interaktive Falldiskussionen geführt, wobei die Teilnehmer über die Chatfunktion aktiv werden können. Die Besonderheit ist in diesem Fall, dass alle Online-Veranstaltungen im Nachhinein von AO-Mitgliedern nach Registrierung auf der Website kostenlos über die Website flexibel abgerufen werden können, auch wenn der eigentliche kostenpflichtige Kurs nicht besucht und ein Zertifikat sowie CME-Punkte nicht erworben werden können. Das Curriculum orientiert sich dabei je nach gewähltem Kurs am AO-Trauma-Curriculum. Wissenschaftlich und inhaltlich verantwortlich ist das Team der AO Trauma Deutschland mit seinem Präsidenten. Die Zielgruppen der Kursangebote wechseln je nach Fortbildungsbereich und reichen von Weiterbildungsassistenten bis zu erfahrenen Operateuren. AO online Campus wird von AO Trauma Deutschland finanziert und ist nicht profitorientiert.

**Figure 2:**
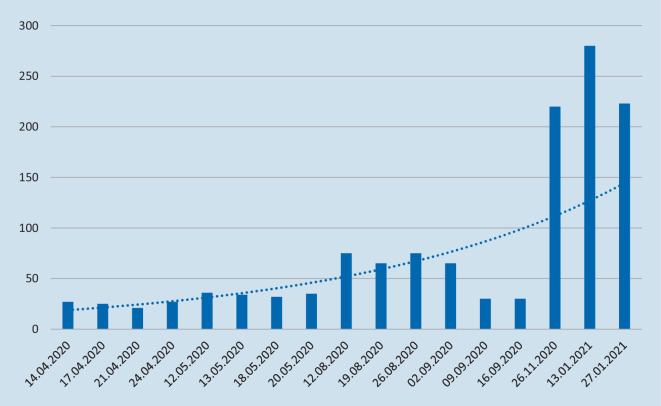

Die Entwicklung der Teilnehmerzahlen der AO-online-Campus-Kurse ist Abb. 2 zu entnehmen. Die einzelnen Termine sind dabei jeweils einem Kurs zugeordnet. Nahmen im Frühjahr 2020 ca. 30 Teilnehmer an den Webinaren teil, stieg die Zahl zum Sommer/zum Herbst auf über 50. Die Traumaspezialkurse als Einzeltermine im November 2020 und Januar 2021 erreichten jeweils über 200 Teilnehmer.

### BVOU Study Club

Der BVOU Study Club ist die Online-Fortbildungsplattform des Berufsverbands für O&U e. V. (BVOU). Seit September 2016 werden regelmäßig Webinare zu verschiedenen orthopädisch-unfallchirurgischen Themen, aber auch zu berufspolitischen oder klinik- und praxismanagementorientierten Themen angeboten. Die Webinare sind nach Registrierung für BVOU-Mitglieder kostenlos; für Nicht-BVOU-Mitglieder besteht die Möglichkeit, ein Jahresabonnement abzuschließen, um auf die Inhalte des BVOU Study Club zuzugreifen. Die Webinare werden wöchentlich (immer mittwochs, in den Abendstunden; ca. 90 min, inklusive Diskussion) live angeboten und sind anschließend flexibel im Webinar-Archiv abrufbar. Nach Bestehen eines Tests zum Thema erhält man ein Zertifikat zur Erlangung von Fortbildungspunkten (CME) bei den Ärztekammern. Zielgruppe der Webinare sind sowohl Weiterbildungsassistenten als auch Fachärzte in O&U. Die Auswahl der Themen und Referenten erfolgt üblicherweise auf Anregung des Vorstandes des BVOU oder durch die Akademie Deutscher Orthopäden (ADO) und folgt inhaltlich keinem festgelegten Curriculum. Die Referenten erhalten üblicherweise kein Honorar. Als Initiative des BVOU arbeitet der BVOU Study Club nicht profitorientiert.

**Figure 3:**
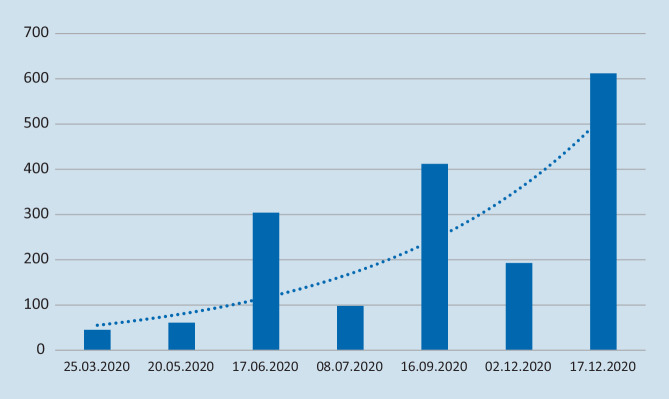

Im Pandemiejahr 2020 stiegen die Teilnehmerzahlen der Study-Club-Webinare tendenziell an, wie Abb. 3 zu entnehmen ist. Nahmen im Frühjahr 2020 zwischen 40 und 60 Teilnehmer aktiv teil, waren es im Juni 2020 (Thema: „Der schwierige Schmerzpatient am Beispiel des unspezifischen Rückenschmerzes“) erstmals über 300 Teilnehmer. Das Webinar zur neuen Heilmittelrichtlinie im Dezember 2020 verfolgten über 600 Teilnehmer live.

### OU TO GO

OU TO GO ist eine reine Online-Fortbildungsplattform und bietet seit März 2020 wöchentliche Fortbildungen aus allen Themenbereichen von O&U an. Die Webinare werden zu festen Zeitpunkten (immer mittwochs, einmal als „early morning session“ und einmal als „late night session“; jeweils 45 min mit anschließender moderierter Diskussion und interaktiver Fragerunde) angeboten. Diese werden nicht aufgezeichnet und sind nicht flexibel abrufbar. Mit einem während der Fortbildung kommunizierten Passwort erhält man ein persönliches Teilnahmezertifikat zur Erlangung von Fortbildungspunkten (CME) bei den Ärztekammern. Die Fortbildungen folgen dabei in Ablauf, Reihenfolge und Inhalt einem zweijährigen, innerhalb der jeweiligen Themen aufeinander aufbauenden Curriculum. Das Team von OU TO GO um den Direktor der Orthopädischen Universitätsklinik der Ruhr-Universität Bochum zeichnet verantwortlich für die Auswahl der Themen und Referenten. Die Ursprünge von OU TO GO gehen auf ein Online-Fortbildungsformat aus dem Fachbereich Gynäkologie zurück, welches bereits seit mehreren Jahren verfügbar ist. Mittlerweile sind weitere Fachbereiche wie die Notfallmedizin oder die Pädiatrie ebenfalls in das Konzept eingestiegen und bieten unter dem Dach von MEDIZIN TO GO Onlinefortbildungen an. OU TO GO ist ein Non-profit-Unternehmen und wird durch Spenden finanziert.

**Figure 4:**
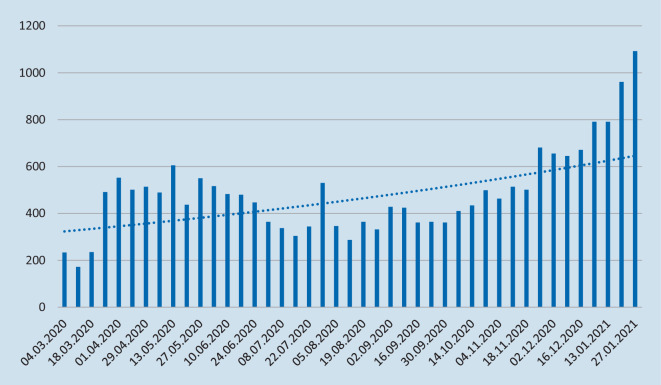

Die erste OU-TO-GO-Session am 04.03.2020 wurde von 233 Teilnehmern gesehen. Im Mai 2020 erreichte das Format mit über 600 Teilnehmern bei der Session „Frakturen der Schulter“ einen ersten Gipfel. In den Sommermonaten nahmen die Teilnehmerzahlen etwas ab, um dann ab November/Dezember 2020 wieder stärker anzusteigen. Mit Beginn des neuen Jahres 2021 nahmen an der Session „Cervikaler Bandscheibenvorfall/Cervikale Spinalkanalstenose“ über 900 und an der Session „Konservative Therapie des Rückenschmerzes“ über 1000 Personen teil. Die Abb. 4 gibt eine grafische Übersicht über die Entwicklung der Teilnehmerzahlen von OU TO GO.

### WebDGU

*WebDGU *„Themen aus der und für die Unfallchirurgie“ ist eine Webinar-Reihe, die seit April 2020 über das Bildungsinstitut für Kompetenz in der Medizin (bikmed), eine Tochtergesellschaft der Akademie der Unfallchirurgie (AUC), angeboten wurde. Die Homepage von bikmed wurde zwischenzeitlich mit dem Kursportal der AUC verschmolzen. Die inklusive Diskussion ungefähr 60- bis 90-minütigen Webinare bzw. Fallvorstellungen werden immer am ersten Mittwoch eines Monats abends live angeboten, die Zugangsdaten zur Live-Session erhält man nach Anmeldung über das Portal. Anschließend sind sie auch ohne vorherige Anmeldung auf dem YouTube-Kanal der AUC flexibel abrufbar. Bei den Live-Veranstaltungen wird die Zuhörerschaft durch Live-Abstimmungen aktiv eingebunden und ist aufgefordert, konkrete Fragen zu stellen, die dann durch die Experten beantwortet und diskutiert werden. Eine CME-Zertifizierung erfolgt mittlerweile regelhaft. Wissenschaftlich und inhaltlich verantwortlich zeichnet die DGU mit ihrem amtierenden Präsidenten, der mit seinem Team auch Themen und Referenten/Experten auswählt. WebDGU wird durch die AUC finanziert und ist nicht profitorientiert. Die Teilnahme am Live-Seminar ist nach Registrierung beim BIKmed kostenlos, und die Aufzeichnungen sind anschließend über YouTube frei verfügbar.

**Figure 5:**
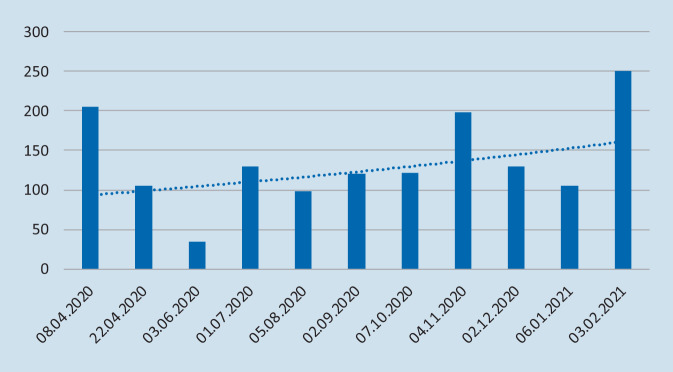

Die erste Session der WebDGU-Reihe war, wie die beiden, folgenden noch mit direktem Bezug zur Pandemie gehalten und wurde von 205 Teilnehmern besucht. In den Sommermonaten nahm die Teilnehmerzahl ab, um dann abgesehen von kleineren Schwankungen im zeitlichen Verlauf recht konstant, um die 100 Teilnehmer zu erreichen. Zum Herbst nahm die Teilnehmerzahl zu; im November 2020 nahmen fast 200 Personen an einem Webinar teil, im Februar 2021 250. Die Entwicklung der Teilnehmerzahlen von WebDGU ist in Abb. 5 dargestellt.

## Diskussion

In der vorliegenden Arbeit konnte gezeigt werden, dass es im zeitlichen Zusammenhang mit der COVID-19-Pandemie zu Veränderungen in der deutschen Fort- und Weiterbildungslandschaft des Faches O&U gekommen ist. Das Angebot von Webinaren und deren steigende Teilnehmeranzahl korrelieren im untersuchten Zeitraum positiv mit dem Andauern der Pandemie. Dieser Trend deckt sich mit Erfahrungsberichten aus den Fachverbänden von O&U. Durch diese Arbeit sind erstmalig konkrete Zahlen offengelegt worden.

Die generelle Entwicklung lässt sich mit Ergebnissen einer gesamtdeutschen Studie der Unternehmensberatung McKinsey und des Stifterverbandes zu Online-Fortbildungsangeboten in Unternehmen in Einklang bringen [9]. In der ärztlichen Weiterbildung lassen sich fächerübergreifend positive Reaktionen auf die veränderte Situation beobachten. Generell werden dem E‑Learning in Deutschland eine positive Relevanz und ein Anstieg von Nutzerzahlen vorhergesagt [10–12]. Eine internationale Befragung in der Dermatologie prophezeit eine nachhaltige Nutzung von E‑Learning [13]. In der Urologie gab es bereits lange vor der COVID-19-Pandemie erfolgreiche Angebote von E‑Learning, welche vermehrten Zulauf erfuhren [14, 15]. In einer Einzelauswertung konnte die Society of Urological Surgery einen konkreten Anstieg der Nutzerzahlen ihrer Webinar-Reihe in der COVID-19-Pandemie benennen [16]. In der plastischen Chirurgie konnte eine Online-Abfrage die gesteigerte Verfügbarkeit von E‑Learning-Angeboten während der COVID-19-Pandemie darstellen [17]. Auch die zukünftige Bereitschaft zur Nutzung von Webinaren wurde in Umfragen bejaht [18]. In der wissenschaftlichen Literatur dominieren fächerübergreifend Online-Umfragen zum Nutzungsverhalten von E‑Learning ohne die quantitative Offenlegung konkreter Angebote.

Zusammenfassend hat die Nachfrage von E‑Learning in der Weiterbildung von der Bedarfsseite durch die COVID-19-Pandemie einen großen Schub erhalten, [8], wobei man sich bewusst machen sollte, dass der Oberbegriff „E-Learning“ eine Vielzahl verschiedener Formate zusammenfasst. Wie nachhaltig dieser Effekt ist, bleibt abzuwarten. Ein gewisser Anteil könnte von Coping-Strategien in der sozialen Isolation beeinflusst worden sein [6]. Aus dem studentischen Umfeld lassen sich kritische Stimmen zum E‑Learning nicht überhören. Es wird z. B. über eine Erhöhung des Workload, einen Qualitätsverlust der Inhalte und eine Vereinsamung vor dem Bildschirm berichtet [19]. Es gibt jedoch auch Ansätze, um E‑Learning durch gezielte Maßnahmen stressärmer und attraktiver zu gestalten [20]; in diesem Zusammenhang wäre sicherlich auch eine Auswertung der Nutzerevaluationen und deren Vergleich in Abhängigkeit von Anbieter und Format von Interesse.

Zur Motivation von Arbeitnehmern kann es für Unternehmen sinnvoll sein, das E‑Learning-Angebot auszuweiten [21]. Den Einführungskosten stehen Ersparnisse durch Wegfall von Reisekosten entgegen. Einen erheblichen Mehrwert digitaler Lernangebote sehen viele Unternehmen darin, dass sich diese gut in den Arbeitsalltag integrieren lassen [5]. Bei einer hohen Krankenhausauslastung und knappen Personalressourcen könnte jedoch auch eine Verdrängung der Fortbildungszeit aus der bezahlten Arbeitszeit in die Freizeit drohen, insbesondere wenn E‑Learning-Angeboten nicht der gleiche Stellenwert wie der Präsenzlehre eingeräumt wird. Eine steigende Akzeptanz könnte hier die Verknüpfung von erworbenen Weiterbildungskenntnissen mit betrieblich erforderlichen Zertifizierungen bieten.

Es kann angemerkt werden, dass die Digitale Agenda der Bundesregierung mit der „Bildungsoffensive für die digitale Wissensgesellschaft“, der „Hightech-Strategie 2025“ und der „Medizininformatik-Initiative“ das hohe Potenzial des digitalen Wandels bereits vor der Pandemie adressiert hat [2]. Der praktische Umgang mit neuen digitalen Möglichkeiten muss jedoch mit langfristigen Strategien erlernt und erprobt werden [22]. Die Vermittlung praktischer chirurgischer Fähigkeiten ist dabei durch Distanzlehre nur schwerlich vorstellbar. Eine Lösung könnte die Kombination von dezentralem E‑Learning mit eigenbetrieblich organisierten praktischen Lerneinheiten im Rahmen von Blended-Learning-Konzepten sein. Alternativ werden adaptierte vollwertige Präsenzveranstaltungen mit neuen Hygienekonzepten erprobt [23, 24]. Nach einer erfolgreichen Durchimpfung und Aufhebung von Dienstreiseverboten wird sich eine Renaissance der Präsenzlehre ergeben. Die Kombination von E‑Learning und Präsenzlehre in sog. Hybridkonzepten ist für O&U perspektivisch eine sehr interessante Option [8, 23–25].

Eine Limitierung dieser Studie ist, dass aus dem gesamten Portfolio des E‑Learning nur das Format der Webinare nichtkommerzieller Anbieter abgebildet wird. Auch internationale Konkurrenzveranstaltungen finden keinen Eingang in dieser Auswertung. Die aus wissenschaftlicher Sicht erforderliche Konzentration auf einen homogenen Themenbereich bildet nicht das gesamte Spektrum der Fortbildungsvielfalt ab. Trotz alledem erscheinen die fachspezifischen nichtprofitorientierten Online-Fortbildungen in Webinar-Form als zentraler Pfeiler der fachlichen Fortbildung in O&U. Die Formate und Organisationsstrukturen der dargestellten Anbieter sind so verschieden, dass sich eine zu kleine Fallzahl für eine sinnvolle vergleichende statische Auswertung ergibt. Daher können die Anbieter nicht in konkurrierend vergleichender Manier beurteilt werden, sondern können nur jeweils an der eigenen Entwicklung gemessen werden. Daher ergibt sich eine rein deskriptive Auswertung. Des Weiteren ist es nicht sinnvoll, rechnerische Vergleiche zu der Zeit vor der Pandemie darzulegen, da sich das eigentliche Angebot von Webinaren überwiegend erst während der Pandemie entwickelt hat. Sehr interessant wäre unter diesem Gesichtspunkt die Entwicklung des Webinar-Angebotes in der Post-COVID-Phase. Leider wurden von den Anbietern keine Daten zu internen Evaluationen zur Verfügung gestellt. Dies wird mit dem Schutz der Referenten begründet und ist zwar bedauerlich, aber durchaus nachvollziehbar.

Das gesteigerte Angebot der Webinare fußt auf unterschiedlichen Interessen. Bei den Anbietern BVOU Study Club und OU TO GO wurden die vorbestehenden Angebote intensiviert. Durch AO online Campus sollten initial die ausgefallenen Kurse kompensiert werden, was in einem pandemietauglichen Webinar-Format gründete. Bei der DGOU mit der WebDGU wurde als Reaktion auf die Einschränkungen der Pandemie ein neuartiges Angebot etabliert. Die verschiedenen Ziele der Akteure erschweren eine Vergleichbarkeit.

Zusammenfassend konnte gezeigt werden, dass es aus dem Inneren der O&U heraus zu einem Umbruch im Bereich Webinare gekommen ist. Auch wenn in der Pandemie bisher Erfolge mit Webinaren verzeichnet wurden, müssen sich die Anbieter auch zukünftig um Nutzer bemühen. Neue digitale Angebote können ganze Branchen als „disruptive technologies“ umwandeln [26]. Das konnte in den letzten Jahren im Bereich Hotelgewerbe, Taxiwesen oder Onlineshopping beobachtet werden. Daher ist eine gute Positionierung der Fachverbände auf dem Markt der Fortbildungsangebote sehr wichtig. Es bleibt zu hoffen, dass finanzielle Ressourcen, Lobbyinteressen und Hierarchiedenken den Umbruch nicht behindern. Die CME-Zertifizierung von erworbenen Weiterbildungskenntnissen könnte einen guten Hebel zur Positionierung am Markt bieten.

Es besteht die Hoffnung auf einen langfristigen synergistischen Effekt von Angebot und Nachfrage in der Digitalisierung. Für die zukünftige Entwicklung müssen politische Entscheidungsträger und auch Berufsverbände weiterhin entsprechende Rahmenbedingungen schaffen.

## Schlussfolgerung

Ziel der vorliegenden Arbeit war eine quantitative Auswertung von Teilnehmerzahlen an deutschsprachigen Online-Fortbildungsangeboten mit Webinar-Charakter aus O&U im zeitlichen Zusammenhang mit der COVID-19-Pandemie. Während OU TO GO und der BVOU Study Club bereits vor der Pandemie auf E‑Learning-Formate spezialisiert waren, wurde das Kursangebot der AO durch den neu etablierten AO online Campus durch E‑Learning-Formate erweitert und das digitale Angebot WebDGU ganz neu konzipiert. Es konnte gezeigt werden, dass die Anzahl von Webinaren als auch deren Teilnehmerzahlen im untersuchten Zeitraum anstiegen. Die Ergebnisse dieser Studie lassen auf eine positive Zukunft der digitalen Fort- und Weiterbildung in O&U im Webinar-Format hoffen.
